# Association between vegetable consumption and calf venous compliance in healthy young adults

**DOI:** 10.1186/s40101-020-00231-z

**Published:** 2020-08-12

**Authors:** Anna Oue, Yasuhiro Iimura, Kotose Maeda, Takahiro Yoshizaki

**Affiliations:** 1 Faculty of Food and Nutritional Sciences, Toyo University, 1-1-1 Izumino, Itakura-machi, Ora-gun, Gunma, 374-0193 Japan; 2 Graduate School of Food and Nutritional Sciences, Toyo University, Gunma, 374-0193 Japan

**Keywords:** Blood pressure, Green/yellow vegetables, Nitric oxide, Venous distensibility

## Abstract

**Background:**

Venous compliance decreases with aging and/or physical inactivity, which is thought to be involved partly in the pathogenesis of cardiovascular disease such as hypertension. This suggests that it is important to maintain high venous compliance from a young age in order to prevent cardiovascular disease. Both nutrient and exercise could play an important role in the improvement and maintenance of vascular health. Indeed, habitual endurance exercise is known to improve the venous compliance, although little is known about the effect of diet on venous compliance. Considering that higher consumption of vegetables could contribute to the arterial vascular health and the decreased blood pressure, it is hypothesized that venous compliance may be greater as vegetable intake is higher. Thus, the purpose of this study was to clarify the association between vegetable intake and venous compliance in healthy young adults.

**Methods:**

Dietary intake was assessed in 94 subjects (male: *n* = 44, female: *n* = 50) using a self-administered diet history questionnaire (DHQ). Intakes of nutrients and food groups that were obtained from the DHQ were adjusted according to total energy intake using the residual method. Based on the adjusted intake of food groups, total vegetable intake was calculated as the sum of green/yellow and white vegetables consumed. Calf volume was measured using venous occlusion plethysmography with a cuff deflation protocol. Calf venous compliance was calculated as the numerical derivative of the cuff pressure–calf volume curve. In addition, circulatory responses (heart rate and systolic and diastolic blood pressure) at resting and maximal oxygen uptake were assessed in all subjects.

**Results:**

Mean value of total vegetables intake was 162.2 ± 98.2 g/day. Simple linear regression analysis showed that greater venous compliance was significantly associated with higher total vegetable consumption (*r* = 0.260, *P* = 0.011) and green/yellow vegetable intake (*r* = 0.351, *P* = 0.001) but not white vegetable intake (*r* = 0.013, *P* = 0.902). These significant associations did not change in the multivariate linear regression models which were adjusted by sex and maximal oxygen uptake.

**Conclusion:**

These findings suggest that higher consumption of vegetables, especially of the green/yellow vegetables, may be associated with greater venous compliance in young healthy adults.

## Background

Aging and physical inactivity cause the decrease in venous compliance as well as arterial compliance [1–4]. Veins are highly compliant vessels that contain 60–70% of total blood volume at rest. The changes in venous capacitance and/or venous compliance with physiological stresses cause the adequate transfer of blood from the vein to the heart, so that cardiac output and blood pressure maintains [5]. This suggests that attenuated venous compliance could have an adverse influence on cardiovascular health. Indeed, the decreased venous compliance has been observed in hypertensive humans [6, 7] and animals [8–10], and the elevation of venous vascular stiffness is thought to be involved partly in the pathogenesis of hypertension. Therefore, it is important to maintain high venous compliance from a young age in order to prevent cardiovascular disease.

Both nutrient and exercise could play an important role in the improvement and maintenance of vascular health. Indeed, habitual endurance exercise is known to increase venous compliance [1, 2, 11–13]. However, little is known about the effect of diet on venous compliance. High consumption of plant-based foods is associated with a reduced risk of cardiovascular disease [14–16]. In addition, the increased consumption of fruit and vegetables produced significant improvement in endothelium-dependent arterial vasodilation in hypertensive subjects [17]. These suggest that high consumption of vegetables could have a beneficial influence on arterial vascular health. Vegetables contain various nutrients, non-nutrients (e.g., fiber, vitamin C, vitamin K, magnesium, and potassium) and phytochemicals (e.g., flavonoids, carotenoids, nitrates, and organosulfur compounds), which have many physiological functions [18, 19]. In particular, the antioxidant, the anti-inflammatory activity, and the increased nitric oxide (NO) synthesis are thought to be possible mechanisms for improvement of cardiovascular and arterial vascular health [20–23]. For example, green leafy vegetables contain many amounts of nitrate that can increase the NO level via the enterosalivary nitrate-nitrite-NO pathway [24]. Furthermore, it has been reported that the consumption of plant-derived bioactive nitrates improved the flow-mediated arterial dilation [25–32] and the pulse wave velocity [29, 31–33]. Considering that NO activity could also contribute to the control of venous tone [34–38], higher consumption of vegetables, especially green/yellow vegetables, could be expected to affect venous compliance.

Thus, the purpose of this study was to investigate the association between vegetable consumption and venous compliance in young healthy adults. It is hypothesized that the consumption of vegetables, especially green/yellow vegetables but not white vegetables, would positively correlate to venous compliance.

## Methods

### Subjects

Ninety-eight young healthy subjects with no overt chronic disease volunteered to participate in this study. All subjects were nonsmokers and not taking any medications. In addition, subjects refrained from caffeine, alcohol, and exercise for 24 h and food intake for 2 h before each experiment. The study was approved by the Human Ethics Committee of Toyo University (approval no. TU2017-006) and was conducted in accordance with the Declaration of Helsinki except for registration in a database. All subjects provided written and verbal informed consent after receiving a detailed explanation of the purpose, procedures, and risks of the study.

### Study design

Subjects visited the experimental laboratory on two occasions. At the first visit, they completed a self-administered diet history questionnaire (DHQ) that assessed their intake of nutrients and food groups. Any mistakes or inconsistencies in responses to the questionnaire items were followed up by well-trained researchers. At the second visit, circulatory responses, venous vascular properties, and maximal oxygen uptake ($\dot{\mathrm{V}}$O_2max_) were measured in this order.

### Measurements

#### Intake of nutrients and food groups

Habitual dietary intake during the previous 1 month was assessed using the DHQ [39, 40]. This questionnaire assesses eating behavior, the amount of 150 kinds of foods consumed, and the amount of 40 kinds of nutrients consumed per day. Data obtained from the DHQ for intake of nutrients and food groups were adjusted according to total energy intake using the residual method [41]. Based on the adjusted intake of food groups, total vegetable intake was calculated as the sum of green/yellow and white vegetables consumed. Energy intake derived from the DHQ has been reported to be moderately correlated with energy expenditure calculated using the double-labeled water method (correlation coefficient: 0.42 for males and 0.37 for females) [42]. Moreover, energy-adjusted vegetable intake determined from the DHQ has also been reported to be moderately correlated with semi-weighted dietary records (correlation coefficient: green and yellow vegetables, 0.32 for males, 0.47 for females; other vegetables, 0.43 and 0.48, respectively) [39].

#### Circulatory response

Systolic (SBP) and diastolic (DBP) blood pressure and heart rate (HR) at rest were measured using an automated sphygmomanometer with the subject in the supine position (Tango M2 Stress Test Monitor, SunTech Medical Inc., Morrisville, NC). The double product (DP) was calculated as the product of SBP and HR.

#### Vascular properties of the calf

All subjects rested in the supine position for at least 20 min before data acquisition. To measure change in calf volume, a venous collecting cuff was wrapped around the left thigh and a mercury strain gage was placed on the site of maximal calf thickness. The collecting cuff was inflated to 60 mmHg for 8 min, after which the cuff pressure was manually reduced from 60 to 0 mmHg at a rate of 1 mmHg/s according to a previously described cuff deflation protocol [43]. Calf volume during cuff inflation and deflation was measured noninvasively using a venous occlusion plethysmography (Hokanson, EC6, D. E. Hokanson, Bellevue, WA). A rapid increase in limb volume was evoked within 3‑4 min of raising the cuff pressure to 60 mmHg, which represented the maximal blood volume stored in the veins, and was followed by a slower fairly linear volume increase caused by net filtration into the extravascular space. We calculated this filtration-dependent increase using the model developed by Skoog et al. [44] and corrected for the limb volume during inflation and deflation of cuff pressure.

Using the corrected calf volume curve, we assessed the vascular properties of the calf venous system as follows. The relationship between cuff pressure and change in calf volume (i.e., the pressure–volume curve) was generated from the data points between 10 and 60 mmHg during the cuff deflation protocol. To avoid any a priori assumptions about the pressure (P)–calf volume (V) curve and to obtain a physiologic venous compliance curve, venous compliance was calculated as the numerical derivative of each pair of pressure–calf volume data points using the following equation [12, 45].

$$ {\mathrm{V}\mathrm{enous}\ \mathrm{compliance}}_{Pi}=\frac{{\mathrm{V}}_i-{\mathrm{V}}_{i-5}}{{\mathrm{P}}_i-{\mathrm{P}}_{i-5}},\mathrm{where}\ 15\le i\le 60 $$

Venous compliance at 20 mmHg of cuff pressure was used as the representative value [2, 43]. Venous capacitance was evaluated as the change in limb volume from before the cuff inflation to 8 min of cuff inflation at 60 mmHg. Venous outflow was calculated from the rate of change in limb volume for 1 min during cuff deflation from 60 to 0 mmHg.

#### Maximal oxygen uptake

Subjects were required to undertake an incremental cycling exercise test (2–30 W/min, pedaling frequency 60 rpm) using a cycle ergometer (Aerobike 75XLШ, Konami Sports Life Co., Ltd., Kanagawa, Japan). Ventilatory and gas-exchange parameters were measured breath-by-breath using a computerized metabolic measuring system (Aero Monitor 310S, Minato Medical Science, Osaka, Japan). HR was measured simultaneously using a monitor (Polar F11, Polar, Kempel, Finland) connected to the metabolic measuring system. At least two of the following criteria were used to determine $\dot{\mathrm{V}}$O_2max_: a respiratory gas exchange ratio > 1.05, HR within 10 bpm of the age-predicted maximal HR, and inability to maintain a pedaling frequency of 50 rpm.

### Statistical analysis

Four of 98 participants who were at the extremes of energy intake (< 500 kcal/day or > 3500 kcal/day for women; < 800 kcal/day or > 4000 kcal/day for men) were excluded from the study [46], leaving data for 94 subjects (44 male, 50 female; age 19–23 years) for inclusion in the analysis. Data are expressed as the mean ± standard deviation for continuous variables and as the number (%) for categorical variables. Spearman’s rank correlation coefficients were calculated to examine the relationship between total vegetable intake and subject characteristics. For simple and multivariate linear regressions, venous compliance was used as the dependent variable, and total vegetable intake, green/yellow vegetable intake, and white vegetable intake were used as independent variables. For multivariate linear regression, an extended model with a covariate adjustment approach was used: model 1 = intake of each vegetable group and sex; model 2 = model 1 plus $\dot{\mathrm{V}}$O_2max_. Statistical analysis was performed using the SPSS software version 19 (IBM Corp., Armonk, NY). A *P* value of less than 0.05 was considered statistically significant.

## Results

Mean value of total vegetable intake was 162.2 ± 98.2 g. In addition, venous compliance, venous capacitance, and venous return in the calf were 0.087 ± 0.030 mL/100 g tissue/mmHg, 2.93 ± 0.88 mL/100 g tissue, and 2.98 ± 0.79 mL/100 g tissue/min, respectively. Total vegetable intake was higher in female than male (*P* < 0.05), and had significant negative association with height, weight, SBP, and DP (*P* < 0.05, Table 1). Furthermore, total vegetable intake had a significant positive relationship with intake of protein, fat, potato, green/yellow vegetables, white vegetables, mushrooms, seaweed, fish and shellfish, beans, fruits, and sugar-sweetened beverages (*P* < 0.05, Table 2). There was a significant negative association between total vegetable intake and carbohydrate or grain intake (*P* < 0.05, Table 2).

**Table 1:** Subject characteristics and Spearman’s correlation coefficient (*r*) values between total vegetable intake and these characteristics

		*r*	*P* value
Age, years	20.2 ± 1.1	0.020	0.846
Height, cm	164.2 ± 8.1	−0.205	0.047
Weight, cm	56.6 ± 8.3	−0.211	0.041
BMI	20.9 ± 2.3	−0.108	0.300
Sex			
Male	44 (46.8)	0.305	0.003
Female	50 (53.2)		
Maximal oxygen uptake, mL/min/kg	38.4 ± 7.0	−0.063	0.546
Systolic blood pressure, mmHg	123 ± 12	−0.304	0.003
Diastolic blood pressure, mmHg	62 ± 7	−0.135	0.194
Heart rate, bpm	65 ± 12	−0.148	0.155
Double product, mmHg×bpm	7916 ± 1461	−0.264	0.010

Values are presented as the mean ± standard deviation for continuous variables and as the number (%) for categorical variables

**Table 2:** Dietary intake and Spearman’s correlation coefficient (*r*) values between total vegetable intake and dietary intake variables

		*r*	*P* value
Energy intake, kcal/day	1933 ± 598	−0.045	0.667
Nutrient intake, g			
Protein	64.6 ± 11.2	0.450	< 0.001
Fat	59.9 ± 14.2	0.442	< 0.001
Carbohydrate	282.2 ± 39.3	−0.407	< 0.001
Food group intake, g			
Grains	490.4 ± 170.8	−0.462	< 0.001
Potato	23.6 ± 21.2	0.361	< 0.001
Nuts and seeds	1.0 ± 1.6	0.106	0.309
Green/yellow vegetables	83.1 ± 71.0	0.815	< 0.001
White vegetables	79.1 ± 50.7	0.747	< 0.001
Mushrooms	7.4 ± 9.2	0.413	< 0.001
Seaweed	10.3 ± 12.2	0.395	< 0.001
Fish and shellfish	44.2 ± 39.7	0.420	< 0.001
Meats	84.1 ± 41.0	0.160	0.124
Eggs	34.0 ± 28.4	−0.073	0.484
Beans	56.0 ±50.1	0.337	0.001
Milk and milk products	134.4 ± 170.4	0.202	0.051
Fruit	127.2 ± 167.8	0.248	0.016
Confectioneries	73.7 ± 45.9	0.017	0.875
Sugar-sweetened beverages	719.7 ± 705.4	0.218	0.035
Alcoholic beverages	26.9 ± 53.5	−0.117	0.261

Values are presented as the mean ± standard deviation for continuous variables. Data were adjusted for energy intake using the residual method

Table 3 shows the standardized coefficients for the relationships between venous compliance and total vegetable intake, green/yellow vegetable intake, and white vegetable intake in multivariate linear regression analysis. In the unadjusted crude model, higher total vegetable intake and green/yellow vegetable intake were significantly associated with greater venous compliance (both *P* < 0.05) but white vegetable intake was not. The significance of these results did not change in the multivariate linear regression models (1 and 2).

**Table 3:** Standardized coefficients (*β*) for total, green/yellow, and white vegetable intakes and venous compliance in multivariate linear regression

	*β*	*P* value
Total vegetables		
Crude	0.260	0.011
Model 1^†^	0.245	0.020
Model 2^‡^	0.205	0.046
Green/yellow vegetables		
Crude	0.351	0.001
Model 1^†^	0.339	0.001
Model 2^‡^	0.306	0.003
White vegetables		
Crude	0.013	0.902
Model 1^†^	−0.008	0.941
Model 2^‡^	−0.029	0.777

^†^Adjusted by sex

^‡^Adjusted by sex and maximal oxygen uptake

## Discussion

The aim of this study was to investigate whether a relationship exists between vegetable consumption and venous compliance in young human adults. Simple linear regression analysis showed that higher total vegetable intake and green/yellow vegetable intake, but not white vegetable intake, were significantly associated with greater venous compliance. These relationships did not change in multivariate linear regression analysis, which was adjusted by sex and $\dot{\mathrm{V}}$O_2max_. These findings suggest that higher consumption of vegetables, especially green/yellow vegetables, might increase venous compliance in young healthy adults.

We found a significant association between higher vegetable consumption and greater calf venous compliance (Table 3). To our knowledge, this is the first study to investigate the relationship between vegetable consumption and venous compliance. Vegetables contain abundant nutrients and phytochemicals. Dietary fiber, magnesium, potassium, vitamin K, vitamin C, flavonoids, carotenoids, nitrates, and organosulfur compounds could be beneficial for cardiovascular health [18] via a number of mechanisms, including antioxidant and anti-inflammatory activity, increased production of NO, and changes in gene expression and signaling pathways [20–23, 47]. It is possible that control of venous compliance is influenced by a combination of these mechanisms. However, exactly which of these contributes the most to venous compliance has yet to be investigated.

Another finding in this study was a significant positive correlation of greater venous compliance with intake of green/yellow vegetables but not white vegetables (Table 3). As mentioned earlier, vegetables contain abundant nutrients and phytochemicals, and many recent studies have reported that nitrates, found predominantly in green leafy vegetables, beetroot, and radish, could improve endothelial function [25–32] and arterial stiffness [29, 31–33] in humans. Vegetable-derived nitrates are thought to increase NO via the enterosalivary nitrate-nitrite-NO pathway. NO has many physiological effects, including relaxation of vascular smooth muscle, elevation of regional blood flow, and inhibition of adhesion of platelets and leukocytes [48], which may cohesively improve endothelial function and prevent arterial stiffness. Venous tone is also regulated by NO [34–38]. Therefore, a possible reason for our finding of an association between green/yellow vegetable intake and calf venous compliance may be the nitrate content of these foods.

Some previous studies have been reported that there are “responders” and “non-responders” to the dietary nitrate supplementation [49, 50]. In the present study, the possibility of individual differences for the effect of green/yellow vegetable intake on venous compliance could not be excluded. Although we have no certain idea, the degree of nitric oxide synthase activity might be considered such as the one of reason for individual variation because nitric oxide synthase has been increased by physical activity [51–53]. Indeed, the variability of physical fitness level between subjects in this study was large (e.g., $\dot{\mathrm{V}}$O_2max_: 26.3 ~ 54.8 mL/min/kg). Thus, as physical activity was higher, the production of NO might be greater, so that venous compliance might be also higher even though the green/yellow vegetable consumption was equal.

Our results could have physiological and clinical significance. Changes in venous structure and function have been described in hypertension [6, 8, 54–56]. For example, both venous compliance and capacitance were found to be decreased in hypertensive subjects [6, 54–56]. Furthermore, in our study, calf venous compliance tended to correlate negatively with SBP (males: *r* = −0.294, *P* = 0.052; females: *r* = −0.239, *P* = 0.095) and DP (males: *r* = −0.275, *P* = 0.092; females: *r* = −0.265, *P* = 0.063) in healthy young subjects. Because venous vascular health is likely to deteriorate with age, maintaining high venous compliance from young adulthood could help to prevent age-related hypertension. Our finding of a possible relationship between consumption of green/yellow vegetables and calf venous compliance in healthy young subjects may pave the way for dietary approaches to prevent or attenuate chronic diseases such as hypertension. Further studies are needed to identify the nutrients and/or phytochemicals in these vegetables that modify venous structure and function.

This study has several limitations. First, the cross-sectional design and the small number of young subjects might have introduced a degree of sampling bias. Therefore, our results cannot be generalized. Second, dietary intake was self-reported. However, the questionnaire used in our research has been validated in previous studies [40, 42], and so we consider our findings credible. Third, this study did not consider the potential effects of the menstrual cycle in female subjects. However, given the evidence that venous compliance at rest does not vary with the menstrual cycle [57], menstruation could be expected to have had little effect on venous compliance in our study. Finally, although we adjusted sex and maximal oxygen uptake in the multivariate linear regression, there could still be potential residual confoundings such as the use of mouthwash and the diets which could affect the venous compliance. However, since we did not control the diet and the mouthwash at least 3 days before the venous compliance assessment, the acute effects of these factors on venous compliance, if any, cannot be excluded completely in this study. Thus, further study would be needed to investigate the effect of vegetable consumption on venous compliance under the experimental condition that the diet and the mouthwash are controlled.

## Conclusions

This study investigated the relationship between vegetable consumption and calf venous compliance in young healthy adults. Our findings suggest that a higher intake of vegetables, especially of the green/yellow vegetables, might increase the venous compliance in this population.

## Data Availability

All data generated or analyzed during this study are included in this published article.

## Abbreviations

DBP: Diastolic blood pressure
DHQ: Diet history questionnaire
DP: Double product
HR: Heart rate
NO: Nitric oxide
SBP: Systolic blood pressure
$\dot{\mathrm{V}}$O_2max_: maximal oxygen uptake
